# RORα overexpression reduced interleukin‐33 expression and prevented mast cell degranulation and inflammation by inducing autophagy in allergic rhinitis

**DOI:** 10.1002/iid3.1017

**Published:** 2023-10-17

**Authors:** Wangbo Yu, Jingwei Du, Lijuan Peng, Tao Zhang

**Affiliations:** ^1^ Department of Otolaryngology‐Head and Neck Surgery The First Affiliated Hospital of Jinan University Guangzhou Guangdong China; ^2^ Department of Otolaryngology‐Head and Neck Surgery Affiliated Hospital of North Sichuan Medical College Nanchong Sichuan China; ^3^ Department of Otolaryngology‐Head and Neck Surgery, Nanchong Central Hospital, The Second Clinical Medical College North Sichuan Medical College Nanchong Sichuan China; ^4^ Department of Microbiology and Immunology School of Basic Medical Sciences，North Sichuan Medical College Nanchong Sichuan China

**Keywords:** allergic rhinitis, autophagy, interleukin‐33, mast cell degranulation, retinoid acid receptor related orphan receptor α

## Abstract

**Background:**

Retinoid acid receptor related orphan receptor α (RORα) is a nuclear receptor that along with other bioactive factors regulates cell proliferation, differentiation, and immunomodulation in vivo.

**Aims:**

The objective of this study was to explore the function and mechanism of RORα in allergic rhinitis (AR).

**Materials and Methods:**

Derp1 was used to construct an AR cell model in HNEpC cells, and RORα was overexpressed or silenced in the AR HNEpC cells. Next, LAD2 cells were co‐cultured with the Derp1‐treated HNEpC cells. Additionally, an AR mouse model was established using by OVA, and a RORα Adenovirus was delivered by nebulizing. Pathological tissue structures were evaluated by hematoxylin‐eosin staining, and the levels of RORα, interleukin‐33 (IL‐33), and other proteins were analyzed immunohistochemistry, western blotting, and immunofluorescence staining. IL‐33, IL‐4, IL‐5, and IL‐13 levels were detected using enzyme‐linked immunosorbent assay kits and cell migration was assessed by Transwell assays.

**Results:**

Our data showed that RORα was downregulated in the nasal mucosa tissues of AR patients. Derp1 treatment could cause a downregulation of RORα, upregulation of IL‐33, the induction of NLRP3 inflammasomes, and cell migration in HNEpC cells. Furthermore, RORα overexpression dramatically attenuated IL‐33 levels, NLRP3 inflammasome activity, and the migration of AR HNEpC cells induced with Derp1. Moreover, RORα in AR HNEpC cells could prevent mast cell (MC) degranulation and inflammation by accelerating autophagy, RORα overexpression inhibited MC degranulation and NLRP3‐induced inflammation in the AR model mice. RORα overexpression reduced IL‐33 expression in nasal epithelial cells, and also suppressed MC degranulation and inflammation by promoting autophagy.

**Conclusion:**

RORα inhibits NLRP3 inflammasome in HNEpC, and attenuated mast cells degranulation and inflammation through autophagy in AR.

## Graphical abstract

- RORα overexpression reduced IL‐33 expression in nasal epithelial cells, and also suppressed mast cell degranulation and inflammation by promoting autophagy
- This study revealed the key roles played by RORα in AR.

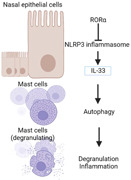

**Abbreviations**
AR: allergic rhinitis
HC: healthy controls
OVA: ovalbumin
RORα: Retinoid acid receptor related orphan receptor α

## 1 INTRODUCTION

Allergic rhinitis (AR) is a frequently diagnosed disease in otorhinolaryngology. It is a noninfectious inflammatory disease of the nasal mucosa that is induced by immunoglobulin E (IgE) following exposure to allergens.^1^ Various immune cells are involved the pathology of AR.^2^ The main clinical symptoms of AR include nasal itching, sneezing, nasal mucosal swelling, and nasal hypersecretion.^3^ Although AR is not a major disease, it affects a wide range of patients. Research has shown that approximately 25% of adolescents and 24% of children experience AR symptoms.^4^ Mast cells (MCs) have been reported to produce Th2‐type cytokines, induce IgE synthesis in B cells, and play a key role in the pathogenesis of AR by self‐activation of the MC‐IgE‐high affinity receptor (FcεRI) cascade.^5^ AR is also associated with asthma, otitis media, sinusitis, allergic conjunctivitis, and other diseases.^6^, ^7^ Although modern medical treatments have a rapid effect on AR, the effect is not long lasting, and the symptoms of AR return. Furthermore, the current treatments for AR can have obvious side effects. Therefore, it is important to explore the pathogenesis of AR and develop new ideas for its treatment.

An allergy is a disease that arises from a combination of both genetic factors and environmental factors.^8^ In view of the complexity of its immunological mechanism, understanding the pathogenesis of an allergy should help to develop new therapeutic targets and hopefully achieve longer lasting and complete efficacy.^9^, ^10^ Studies have shown that elevated levels of interleukin‐33 (IL‐33) in AR patients are related to the development of Th2 and Th17 inflammation; thus providing a new idea for the pathogenesis of AR.^11^, ^12^ IL‐33 is a member of the IL‐1 family of cytokines, which also includes IL‐1β and IL‐18.^13^ IL‐33 is thought to be involved in Th2‐mediated allergic diseases.^14^ Recent studies confirmed that IL‐33 can promote systemic and local Th2 inflammation.^15^, ^16^ Besides, IL‐33 functions as a MC chemoattractant, aiding in their recruitment to specific inflammation sites.^17^ IL‐33 also free up MCs to produce proinflammatory cytokines and mediators, thereby promoting inflammation. Moreover, it encourages the granulation process of MCs leading to degranulation, which further escalates the inflammation process.^18^ Therefore, it is of great significance to further study molecular targets related to IL‐33 to improve AR therapy.

Retinoid acid receptor related orphan receptor α (RORα) is a transcription factor that is expressed in multiple human tissues.^19^ RORα is not only involved in regulating human metabolism, but also plays key roles in chronic inflammation, cellular stress, immune dysfunction, tumorigenesis, and other processes.^20^, ^21^ Furthermore, RORα has been verified to modulate the function of Th17 cells, which are relevant to various autoimmune diseases, such as inflammatory colitis and rheumatoid arthritis.^22^, ^23^ Therefore, we preliminarily speculated that RORα might affect the AR process by regulating IL‐33. However, the role and mechanism of RORα in AR have not been previously reported.

The aim of this study is to investigate the biological function of RORα in nasal epithelial cells, and its role in the relationship constituted by nasal epithelial cells and MCs. In this study, we investigated the underlying function and mechanism of RORα in AR. First, we created AR cell and mouse models, and then used them to further investigate whether RORα could affect the development of AR, including NLRP3 inflammasome formation and cell migration. We also explored the function and mechanism of RORα in MCs, and its effects on degranulation and inflammation. Our study provides some new ideas for improving AR therapy.

## 2 MATERIALS AND METHODS

### 2.1 Tissue samples

Samples of nasal mucosa tissue were obtained from healthy control (HC) subjects and AR patients who were admitted to the Affiliated Hospital of North Sichuan Medical College from June 2020 to December 2021. Each patient and control subject provided their informed written consent for study participation. The study protocol was approved by the Ethical Review Committee of Affiliated Hospital of North Sichuan Medical College (Approval No. 2022ER144‐1). The collected tissues were stored at −80°C or fixed in 4% paraformaldehyde.

### 2.2 Cell culture

Epithelial cells (HNEpC) were purchased from Procell Life Science & Technology Co., Ltd. LAD2 human MCs were obtained from CCTCC. The HNEpC cells were cultured in RPMI‐1640 medium (Sigma) containing 10% fetal bovine serum and 1% Penicillin and Streptomycin. All cells were cultured at 37°C in a 5% CO_2_ atmosphere and 100% humidity.

### 2.3 Cell treatment and co‐culture

HNEpC cells were treated with 0, 5, 10, or 15 μg/mL Derp1 to induce the AR cell model. RORα small interfering RNA (siRNA) (siRNA#1: AGACAAUGACCCAUGAUUGACdTdT, siRNA#2: UUUAUGUGCUCAAGUUGAGACdTdT, and siRNA#3: AGUUUAUGUGCUCAAGUUGAGdTdT), and negative control (NC: GAUAAUGACAGACCCAAUGUAACdTdT) were provided by Genepharma. An RORα overexpression plasmid and empty vector (EV) were purchased from Integrated Biotech Solutions. The HNEpC cells were cultured in six‐well plates and transfected with the siRNAs, NC, RORα overexpression plasmid or EV by using Lipofectamine 3000 reagent (Invitrogen). After 48 h, transfection efficiency was assessed in the transfected cells.

Noncontact co‐culture experiments were performed using Transwell chambers. In the experiments, the upper chamber was inoculated with HNEpC or LAD2 and the lower chamber was inoculated with LAD2 or HNEpC. During the co‐culture, the ratio of the number of cells inoculated in the upper and lower chambers was 1:5. The culture system was placed in an incubator for incubation and subsequent assay experiments were performed.

### 2.4 Experimental animals

Mature BALB/C mice (male, *n* = 24; 6–8 weeks old; weight range = 18–22 g) were purchased from the Animal Center of North Sichuan Medical College. The mice were housed in an SPF environment (temperature = 22°C–24°C; humidity = 55% ± 5%; ad libitum diet; 12 h light/dark cycle). All procedures performed with animals were approved by the Ethics Committee of North Sichuan Medical College (Approval No. 2022ER144‐1).

### 2.5 Construction of the AR mouse model

A total of 24 mice were randomly divided into 3 groups: Sham, AR, AR+RORα. Among them, two groups (16 mice) were used for OVA induction and formed AR animal models. If the mice model showed symptoms such as nose scratching and runny nose, it indicated that the model was successful. Animals without these symptoms were considered experimental failures and were excluded from this experiment. Mice in the AR (including AR+RORα) group (*n* = 16) were injected intraperitoneally with OVA once every other day for 7 times (to induce systemic immune response), and mice in the control group (*n* = 8) were injected intraperitoneally with 0.9% saline according to the same schedule.

Stimulation phase: Starting on day 14, mice in the experimental group were given a 5% OVA nasal drip (20 μL, once/day) for a period of 7 days (to Induce allergic response in the nasal cavity). Treatment efficacy in the experimental group was judged based on symptoms displayed by the mice. The mice in the Sham group were treated with an equal volume of saline as a control group.

The tasks of animal husbandry and experimental data/sample collection are assigned to different individuals. After the experiment was completed, mice were anesthetized with pentobarbital sodium and then euthanized by cervical dislocation, with the criteria for death being the absence of heartbeat and tail flick reflex in the animals.

### 2.6 RT‐qPCR

Total RNA was extracted from processed HNEpC cells using a TRIzol kit (Invitrogen). After purification, the total RNA was used to synthesize complementary DNA with a Bestar™ qPCR RT kit (DBI Bioscience). Next, RT‐PCR was performed using a SYBR Green qPCR Mix kit (Sparkjade). Relative levels of gene expression were calculated using the 2^−ΔΔCt^ method. The primers used in this study was listed as following:

RORα (*Homo sapiens*) forward: ACTCCTGTCCTCGTCAGAAGA

RORα (*Homo sapiens*) reverse: ACTCCTGTCCTCGTCAGAAGARORα (*Mus musculus*) forward: GTGGAGACAAATCGTCAGGAAT

RORα (*Mus musculus*) reverse: TGGTCCGATCAATCAAACAGTTC

GAPDH (*Homo sapiens*) forward: TGTTCGTCATGGGTGTGAAC

GAPDH (*Homo sapiens*) reverse: ATGGCATGGACTGTGGTCAT

GAPDH (*Mus musculus*) forward: AGGTCGGTGTGAACGGATTTG

GAPDH (*Mus musculus*) reverse: GGGGTCGTTGATGGCAACA

### 2.7 Western blotting

The nasal mucosa tissues from each group of mice were collected and ground to a powder. Aliquots of HNEpC and LAD2 cells that had been treated were collected. Total protein was extracted by using ice‐cold RIPA lysis buffer and the total protein concentration in each extract was determined by the BCA method. A 50 μg sample of protein from each extract was separated by sodium dodecyl sulfate–polyacrylamide gel electrophoresis, and the separated protein bands were transferred onto polyvinylidene fluoride membranes (Merck), which were subsequently blocked. Next, the membranes were incubated overnight at 4°C with primary antibodies of RORα (NBP1‐52813, 1:1000; Novus Biologicals), LC3 (PA01524, 1:1000; Boster), beclin 1 (PB9076, 1:1000; Boster), p62 (PB0458, 1:2500; Boster), NLRP3 (27458‐1‐AP, 1:2000; Proteintech), Caspase 1 (81482‐1‐RR, 1:5000; Proteintech), ASC (10500‐1‐AP, 1:4000; Proteintech), and GAPDH (GB15002, 1:2000; Servicebio). After washing, the membranes were incubated with a secondary antibody at room temperature for 1.5 h. The immunostained protein bands were detected by enhanced chemiluminescence (ECL; Thermo). The Grayscale values of the target protein bands were analyzed using ImageJ software.

### 2.8 Immunofluorescence (IF) assay

An aliquot from each group of suspended HNEpC cells (including crawling cells) was inoculated in the wells of a 24‐well plate at a density of 5 × 10^4^ cells/well. After being mounted the cells were washed and fixed with 40 mL/L paraformaldehyde for 30 min, and then permeabilized with 10 mL/L Triton X‐100 for 15 min. The cells were then blocked with 5 g/L bovine serum albumin at RT for 30 min and subsequently incubated with antibody of IL‐33 (12372‐1‐AP, 1:500; Proteintech), RORα (NBP1‐52813, 1:400; Novus Biologicals) or LC3B (PA01524, 1:300; Boster) at 4°C overnight. After washing, the cells were incubated with FITC‐labeled goat antirabbit IgG (1:100) for 60 min at 37°C. After DAPI (Sigma) staining of the nucleus, the cells were blocked with antiquenching agent and observed and photographed under a fluorescence microscope.

### 2.9 Enzyme‐linked immunosorbent assay (ELISA)

Cell supernatants and samples of mouse serum were collected from each group. The levels of IL‐33, β‐hexosaminidase, histamine, tryptase, IL‐4, IL‐5, IL‐13, and IgE in the supernatants and serum samples were detected by using the corresponding ELISA kits according to the manufacturer's instructions.

### 2.10 Transwell assays

HNEpC cells from each group were digested and resuspended, and the concentrations were adjusted to 5 × 10^4^ cells/well. Next, 200 μL of HNEpC cell suspension was added to the upper chamber of a Transwell plate, and 600 μL of complete medium was added to the lower chamber. The cells were then incubated at 37°C for 48 h, and cells in the upper chamber were carefully wiped off with a moistened cotton swab. The migrated cells were fixed with 4% paraformaldehyde and stained with crystal violet. The numbers of cells in five randomly selected fields were counted under a light microscope.

### 2.11 Transmission electron microscope (TEM)

TEM was used to observe the autophagosomes in LAD2 cells. Briefly, groups of LAD2 cells were collected, treated with 2.5% glutaraldehyde, and fixed with 1% osmium acid. After dehydration and permeabilization, the samples were double‐stained with uranyl acetate and lead citrate. The results were observed under a TEM (H‐500; Hitachi).

### 2.12 Hematoxylin‐eosin (H&E) staining

The collected nasal mucosa tissues from HC and AR patients were fixed using formalin solution. Each tissue was incubated in a gradient alcohol series for dehydration and made transparent by treatment with xylene. After immersion wax embedding, the trimmed wax blocks were placed on a paraffin microtome for continuous sectioning at a thickness of 4 μm. Finally, the sections were baked at 60°C, subjected to conventional H&E staining, and observed under a light microscope.

### 2.13 Immunohistochemistry (IHC) staining

Tissue sections were completely submerged in citrate buffer and repeatedly heated three times in a microwave oven. After natural cooling, the sections were washed and closed with 10% normal goat serum at RT for 30 min; after which, they were incubated in a wet box with diluted RORα antibody (NBP1‐52813, 1:400; Novus Biologicals) at 4°C for 12 h. After washing, the sections were incubated with biotin‐labeled goat anti‐rabbit antibody at RT for 30 min. The sections were then washed and subjected to DAB color development. Next, the sections were washed again, treated with DAB and hematoxylin, and sequentially processed for differentiation, dehydration, and transparency. After sealing, RORα expression in the tissues was observed under a microscope.

### 2.14 Statistical analysis

All data were analyzed using IBM SPSS Statistics for Windows, Version 20 software (IBM Corp.). All experiments were independently repeated 3 times and results are expressed as a mean value ± *SD*. One‐way analysis of variance was used to analyze the significance of differences between groups. A *p* < .05 was considered to be statistically significant. The Mann–Whitney *U* test will be used if the data does not follow normal distribution. A 95% confidence interval was used for statistical analysis of the data.

## 3 RESULTS

### 3.1 RORα was downregulated in the nasal mucosa tissues of AR patients

**Figure 1:**
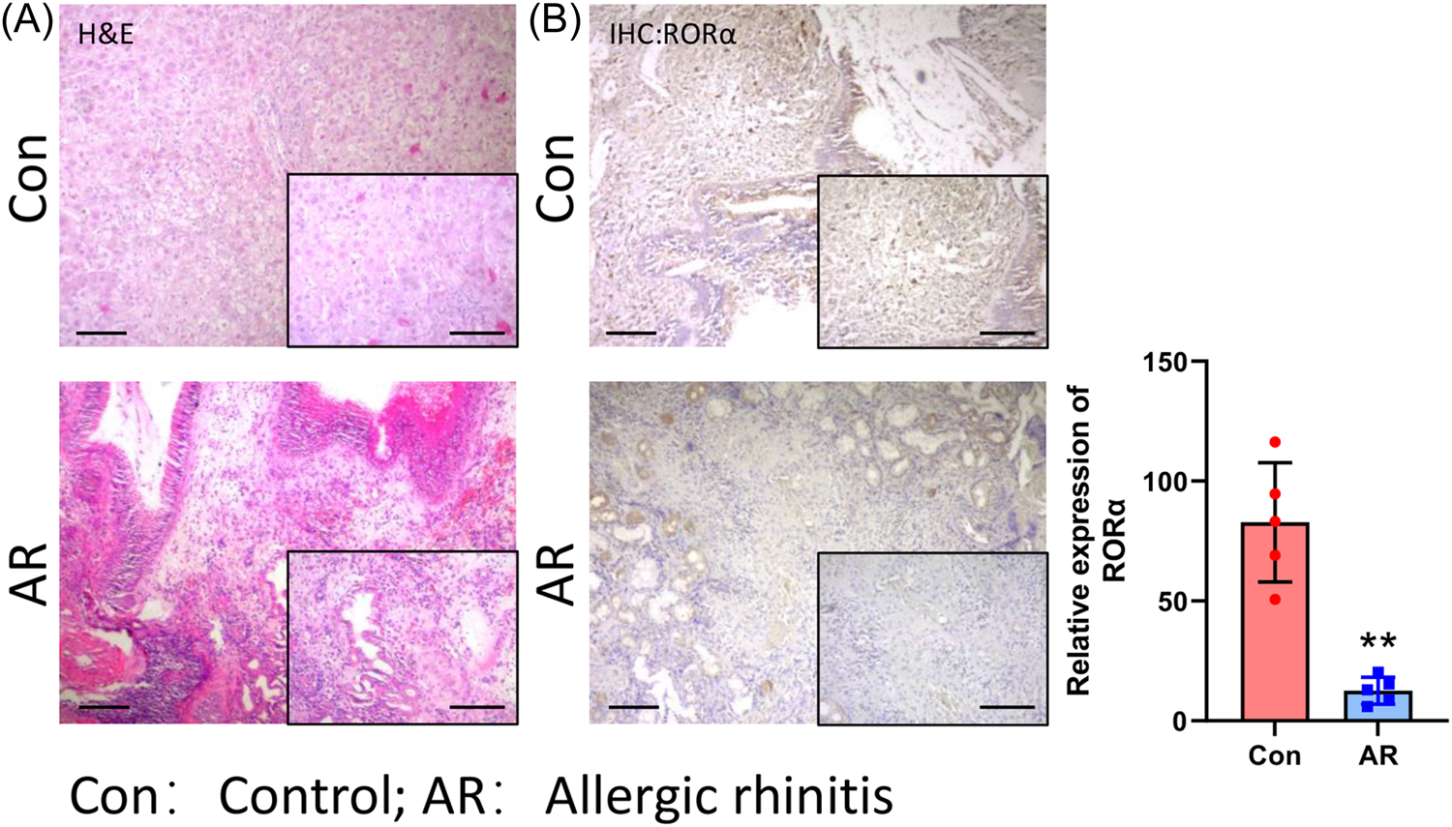
RORα was downregulated in the nasal mucosa tissues of AR patients. (A) Pathological structural changes in the control and AR tissues were observed after H&E staining. (B) IHC assays were performed to assess the localization of RORα in control and AR tissues. Magnification, ×100 or ×200. AR, allergic rhinitis; H&E, hematoxylin‐eosin; IHC, immunohistochemistry; RORα, receptor related orphan receptor α.

To confirm the expression of RORα in AR patients, we first collected samples of nasal mucosa tissues from AR patients and Non‐AR patients (Ctrl). H&E staining showed that in the Ctrl group, the nasal mucosal epithelium was intact with no inflammatory cell infiltration, while in the AR group, varying degrees of epithelial cell destruction and submucosal eosinophil infiltration were observed in the nasal mucosa tissue (Figure 1A). IHC data revealed that RORα expression was downregulated in the nasal mucosa tissues of AR patients relative to its expression in the control subjects (Figure 1B). Overall, we testified the low expression of RORα was found in the nasal mucosa tissues of AR patients.

### 3.2 Derp1 downregulated RORα in HNEpC cells

**Figure 2:**
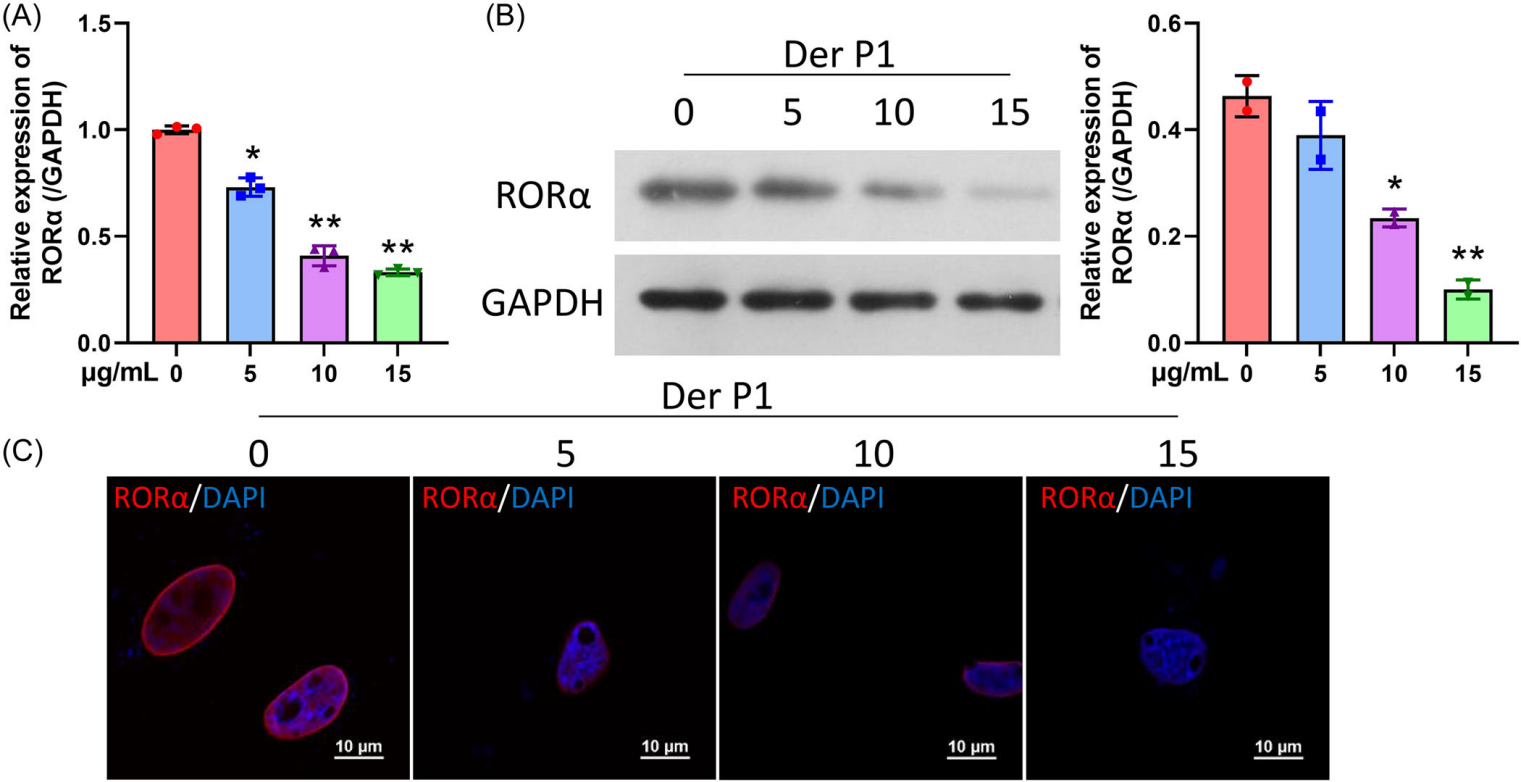
Derp1 markedly downregulated RORα in HNEpC cells. Epithelial cells (HNEpC) were cultured and treated with 0, 5, 10, and 15 μg/mL Derp1 to construct an in vitro model. (A) RT‐qPCR results showed the changes in RORα expression. (B) Western blot assays were performed to evaluate changes in RORα protein expression. (C) The expression and location of IL‐33 in HNEpC cells were evaluated by IF assays. Magnification, ×400; scale bar = 10 μm. *p < .05; **p < .01. IF, immunofluorescence; RORα, receptor related orphan receptor α.

Derp1 was used to construct an AR cell model in HNEpC cells. As shown in Figure 2A, the levels of RORα messenger RNA were markedly lower in the Derp1 treatment groups when compared to those in the cells treated with 0 μg/mL. The Der p1 suppressed RORα expression in a dose‐dependent manner (Figure 2A). Meanwhile, western blotting data also showed that RORα protein levels were significantly reduced in the Derp1 treatment groups (especially in the high concentration group) when compared to those in the control group (Figure 2B). Furthermore, IF results showed that Derp1 could cause a significant downregulation of RORα in HNEpC cells (Figure 2C).

### 3.3 Derp1 upregulated IL‐33 and induced NLRP3 inflammasome formation and HNEpC cell migration

**Figure 3:**
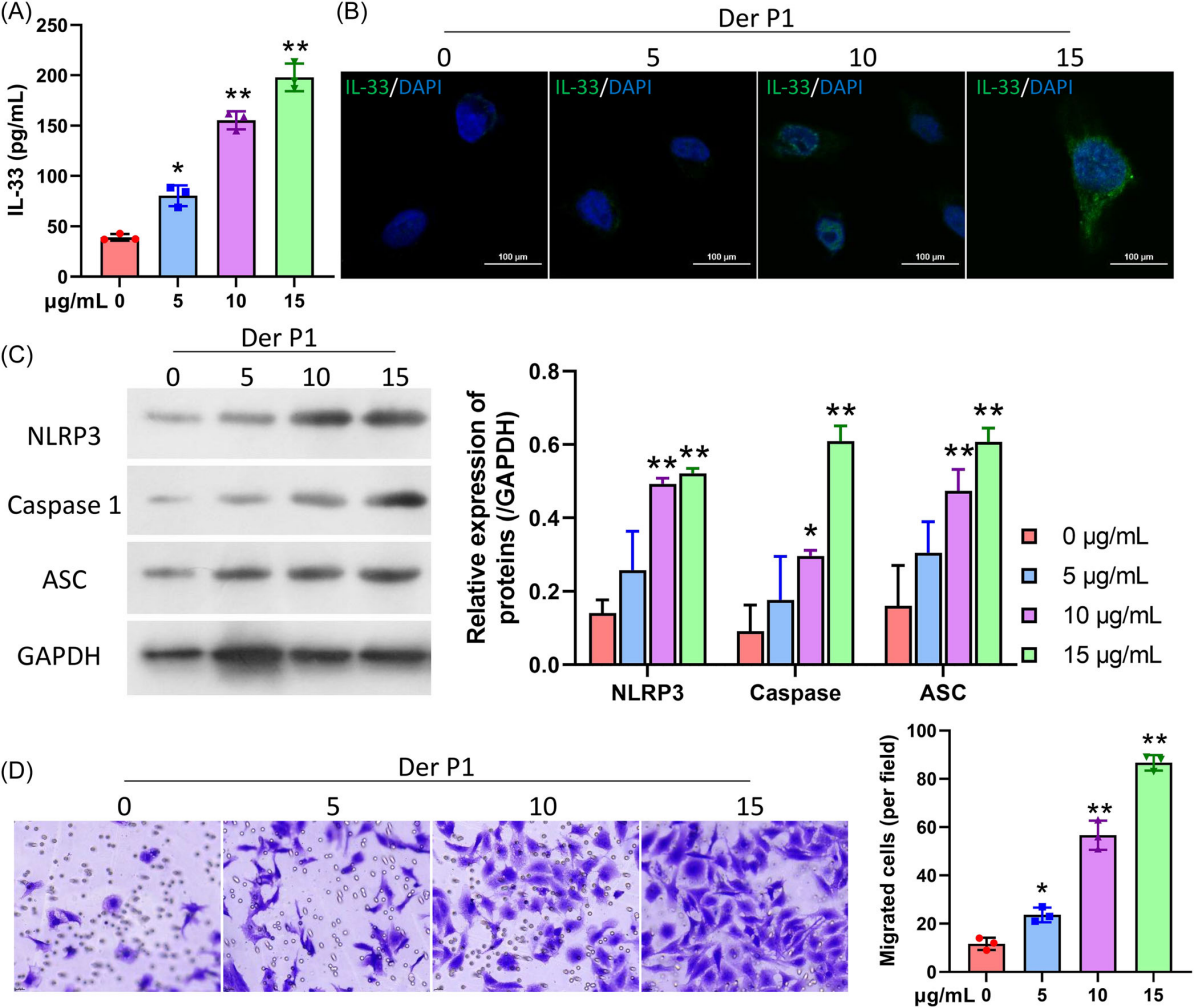
Derp1 upregulated IL‐33, induced NLRP3 inflammasome formation, and stimulated the migration of HNEpC cells. HNEpC cells were treated with 0, 5, 10, and 15 μg/mL Derp1, respectively. (A) ELISA data showed the levels of IL‐33. (B) IF assays were performed to monitor IL‐33 expression. Magnification, ×200; scale bar = 100 μm. (C) Changes in NLRP3, Caspase 1, and ASC protein expression were assessed by western blotting. (D) Transwell assays were conducted to analyze cell migration ability, and the numbers of migrated cells were estimated. Magnification, ×200. *p < .05; **p < .01. ELISA, enzyme‐linked immunoassay; IF, immunofluorescence; IL, interleukin.

Next, we further assessed the changes that occurred in NLRP3 inflammasomes and the migration of AR HNEpC cells induced with Derp1. First, ELISA data showed that the concentrations of IL‐33 were increased in the Derp1 groups (especially in the 15 μg/mL Derp1 group) relative to those in control group (Figure 3A). Similarly, IF data indicated that IL‐33 was predominantly located in the cytoplasm, and treatment with Derp1 could lead to a remarkable decrease of IL‐33 expression (Figure 3B). We then discovered that Derp1 induction could elevate NLRP3, Caspase 1, and ASC expression in HNEpC cells in a dose‐dependent manner (Figure 3C). Transwell data showed that the migration ability of HNEpC cells was dramatically enhanced in the Derp1 groups various with increasing concentration of Der P1 (Figure 3D). Overall, these data showed that NLRP3 inflammasome activity and cell migration were notably enhanced, and IL‐33 levels were increased in the AR HNEpC cells.

### 3.4 Validation of RORα overexpression and knockdown in HNEpC cells

**Figure 4:**
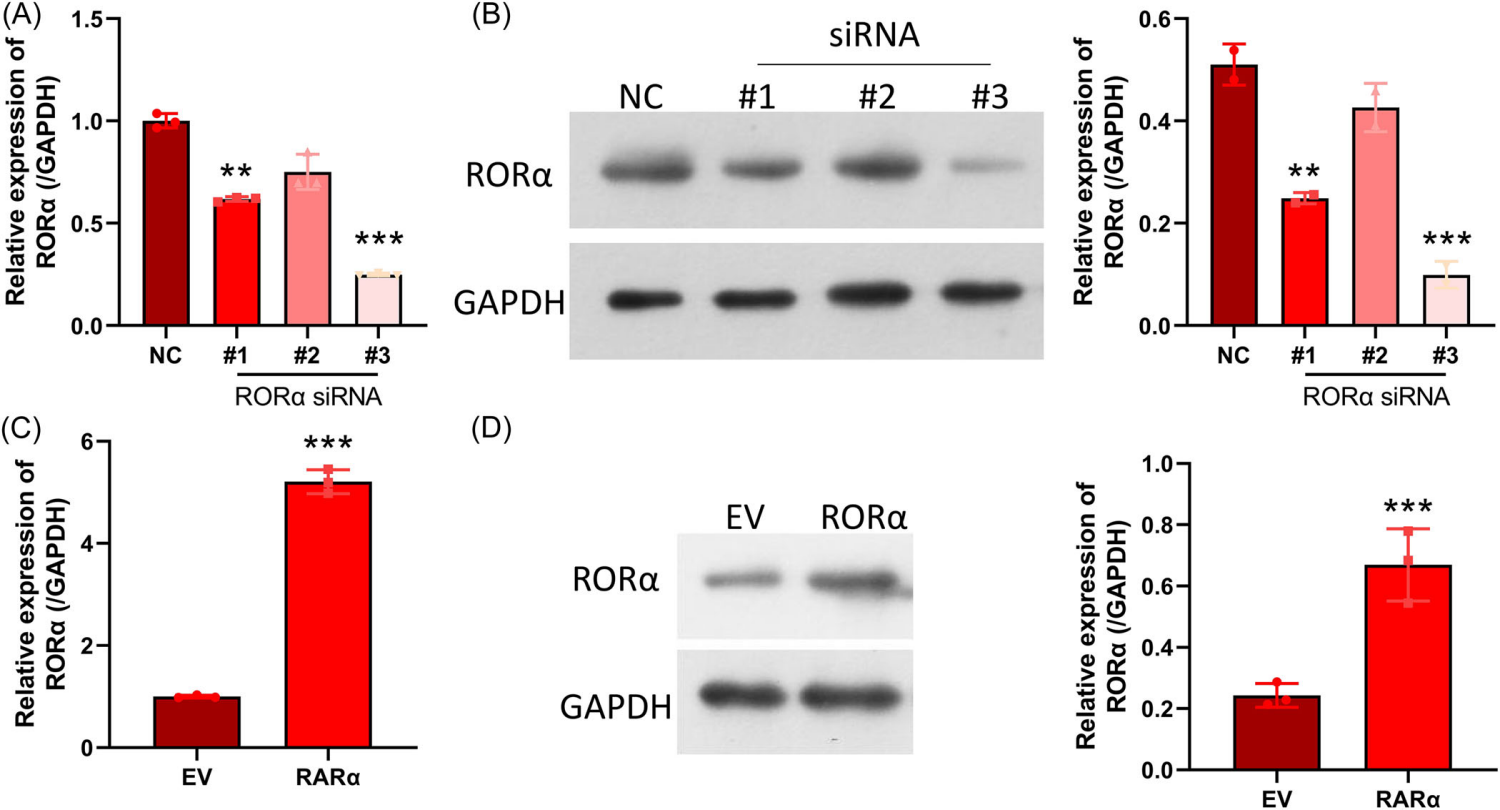
RORα was overexpressed or silenced in HNEpC cells. HNEpC cells were transfected with RORα siRNAs (siRNA#1, siRNA#2, and siRNA#3), and RORα expression was determined by RT‐qPCR (A) and western blotting (B). After transfection with the RORα expression plasmid, the levels of RORα were determined by RT‐qPCR (C) and western blotting (D). **p < .01; ***p < .001. GAPDH, glyceraldehyde 3‐phosphate dehydrogenase; RORα, receptor related orphan receptor α; siRNA, small interfering RNA.

To further determine the potential role of RORα, we knocked down or overexpressed RORα in HNEpC cells. Our data showed that RORα inhibition by transfection with different siRNAs significantly decreased RORα expression in HNEpC cells, and siRNA#3 showed the best knockdown efficiency in HNEpC cells (Figure 4A,B). We also found that RORα overexpression could notably increase RORα expression in HNEpC cells (Figure 4C,D). These results proved that RORα could be successfully overexpressed or silenced in HNEpC cells by transfection.

### 3.5 Overexpression and silencing of RORα in Derp1‐treated HNEpC cells

**Figure 5:**
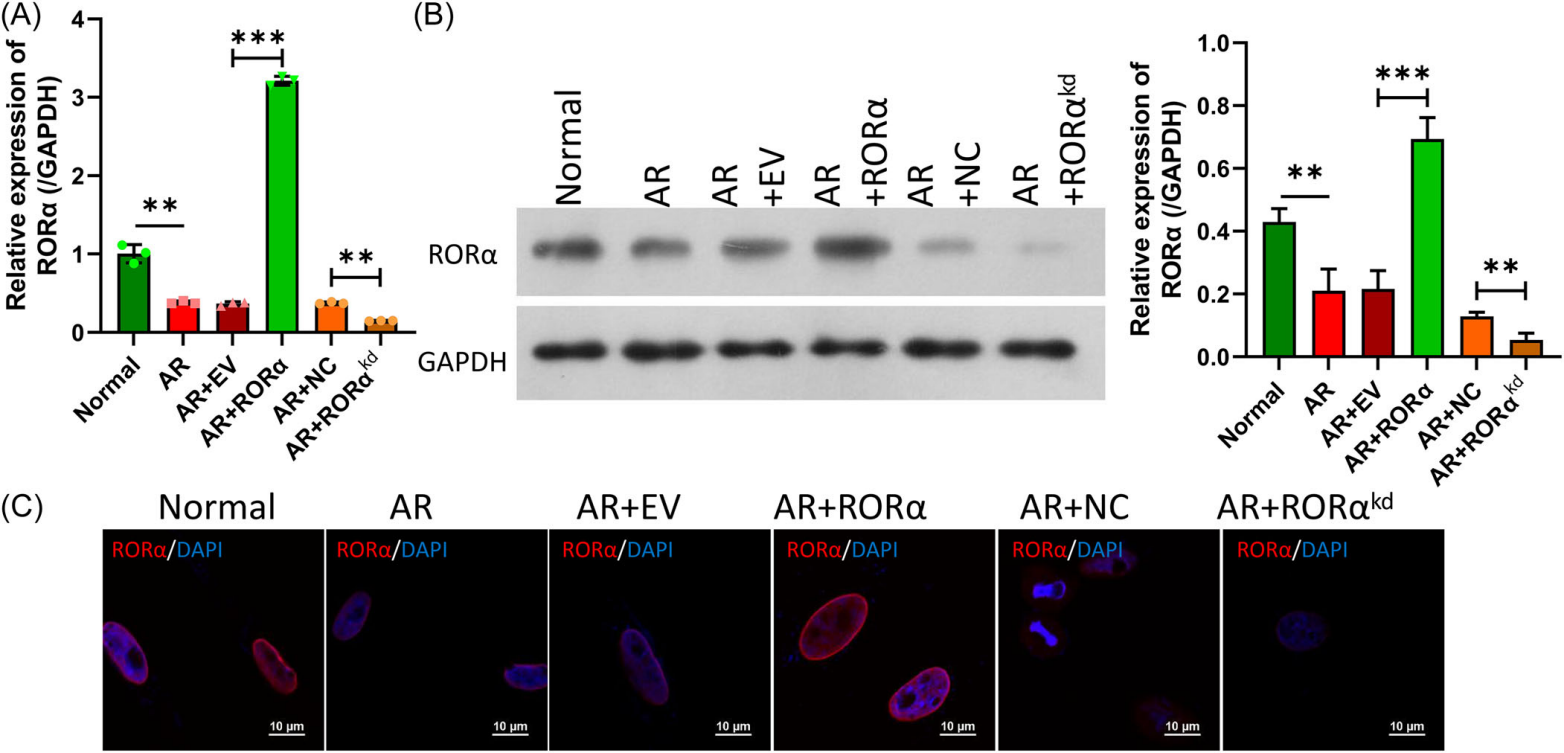
Overexpression or silencing affected RORα in Derp1‐treated HNEpC cells. HNEpC cells were treated with Derp1 (15 μg/mL), and then transfected with the RORα overexpression plasmid or RORα siRNAs, respectively. (A) Changes in RORα expression were determined by RT‐qPCR. (B) RORα protein levels were analyzed by western blotting. (C) IF assays revealed the changes in RORα expression. Magnification, ×400; scale bar = 10 μm. **p < .01; ***p < .001. IF, immunofluorescence; RORα, receptor related orphan receptor α; siRNA, small interfering RNA.

Subsequently, we further transfected the RORα overexpressed plasmid or RORα siRNAs into AR HNEpC cells (cells induced with Derp1). RT‐qPCR data indicated that RORα expression was notably reduced in the AR group when compared to the normal group. Moreover, RORα overexpression could dramatically upregulate RORα expression, and RORα silencing could significantly downregulate RORα expression in AR HNEpC cells (Figure 5A). Likewise, western blot and IF results showed that RORα overexpression elevated the levels of RORα protein expression, and RORα silencing markedly reduced RORα protein expression in AR HNEpC cells (Figure 5B,C).

### 3.6 The effects of RORα on IL‐33 levels, NLRP3 inflammasomes, and the migration of AR HNEpC cells induced with Derp1

**Figure 6:**
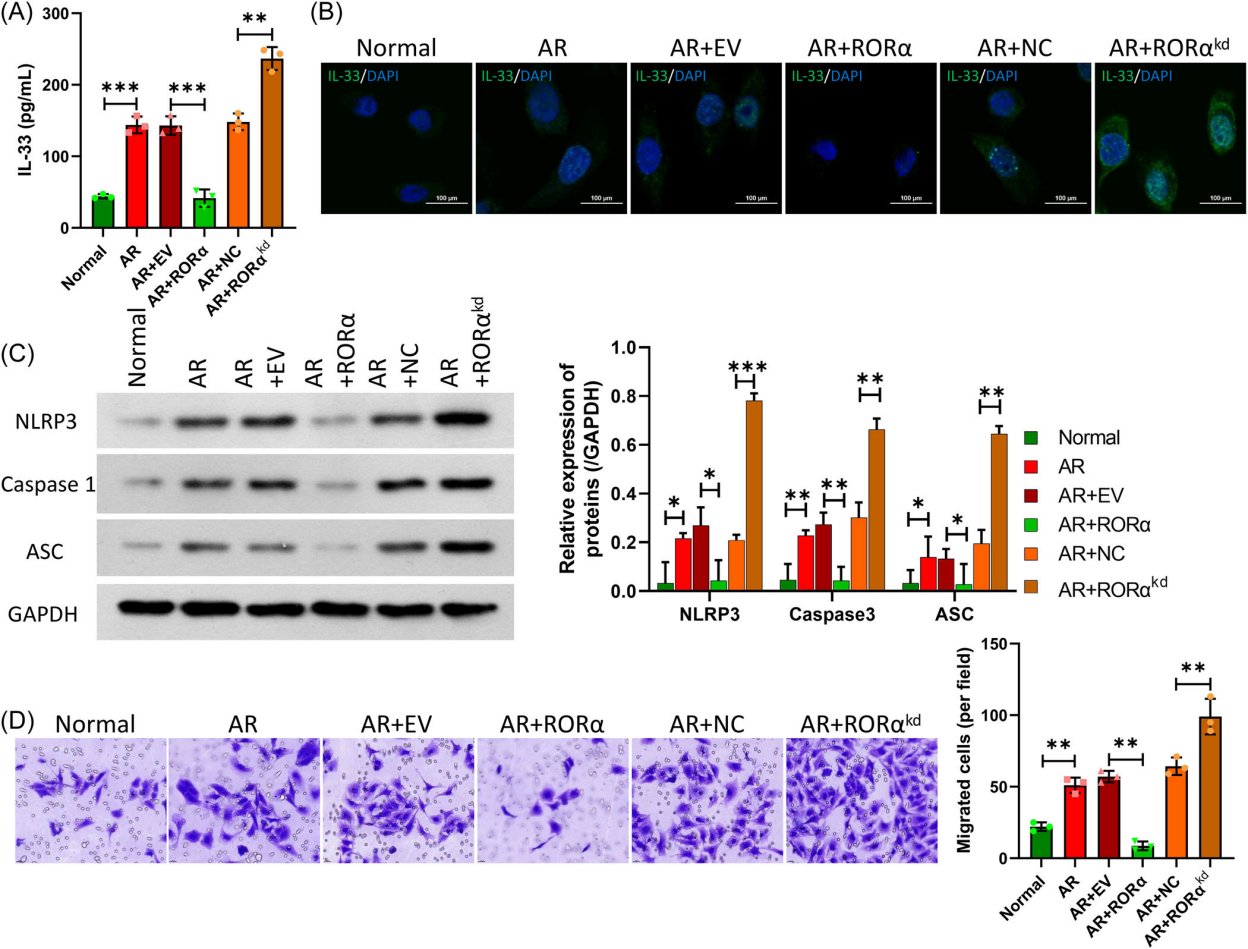
The effects of RORα on IL‐33 levels, NLRP3 inflammasomes, and the migration of AR HNEpC cells induced with Derp1. Transfections were performed to force the overexpression or silencing of RORα in AR HNEpC cells treated with Derp1. (A) ELISA analysis of IL‐33 expression. (B) Changes in IL‐33 expression in each group were confirmed by IF assays. Magnification, ×200; scale bar = 100 μm. (C) Western blotting was performed to detect changes in NLRP3, Caspase 1, and ASC protein expression. (D) Cell migration detected by the Transwell assay, and the numbers of the migrated cells were counted. Magnification, ×200. *p < .05; **p < .01; ***p < .001. ELISA, enzyme‐linked immunoassay; IL, interleukin; RORα, receptor related orphan receptor α.

We explored the effects of RORα overexpression or silencing on IL‐33, NLRP3 inflammasomes, and the migration of AR model cells. ELISA data showed that the levels of IL‐33 were markedly lower in the RORα overexpression group than in the AR group, and the levels of IL‐33 were markedly higher in the AR+RORα silencing group than in the AR group (Figure 6A). IF results also indicated that RORα overexpression caused a decrease in IL‐33 levels, while RORα silencing markedly increased IL‐33 levels in AR HNEpC cells induced by Derp1 (Figure 6B). Western blot data showed that RORα overexpression dramatically downregulated NLRP3, Caspase 1, and ASC protein expression, while RORα silencing notably upregulated NLRP3, Caspase 1, and ASC protein expression in AR HNEpC cells (Figure 6C). Moreover, Transwell data verified that RORα overexpression caused a marked decrease in the migration of AR HNEpc cells, and RORα silencing led to a significant increase in the migration of AR HNEpC cells (Figure 6D). Thus, our data revealed that overexpression of RORα could reduce IL‐33 expression and NLRP3 inflammasome activity in AR HNEpC cells, and also the migration of the cells.

### 3.7 The effects of RORα in AR HNEpC cells on MC degranulation, autophagy, and inflammation in LAD2 cells

**Figure 7:**
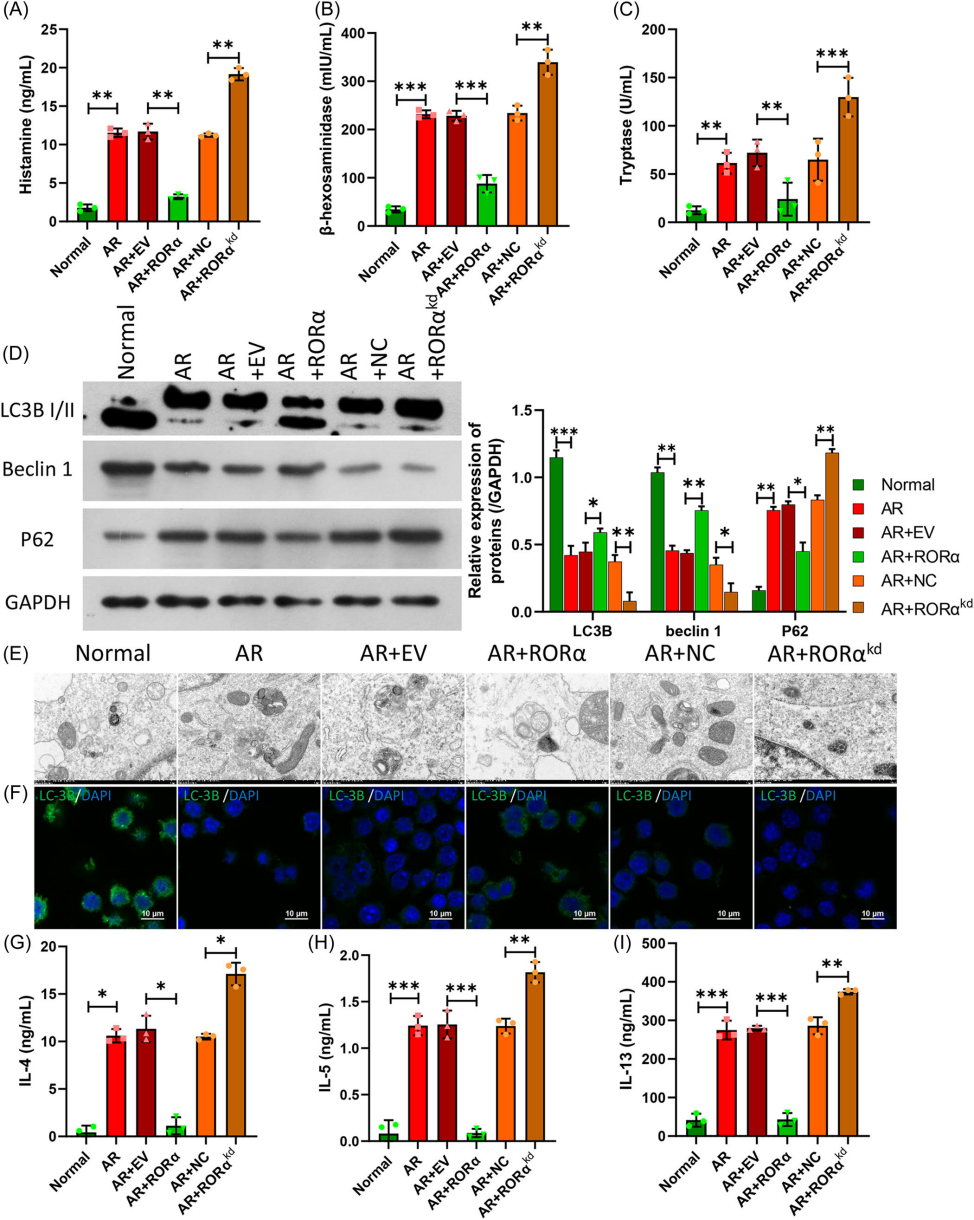
The effects of RORα in AR HNEpC cells on mast cell degranulation, autophagy, and inflammation in LAD2 cells. LAD2 cells were co‐cultured for 24 h with AR HNEpC cells that overexpressed or did not express RORα, respectively. ELISA kits were used to determine the concentrations of β‐hexosaminidase (A), histamine (B), and tryptase (C) in cell supernatants. (D) Changes in LC3B, Beclin 1, and P62 protein expression in LAD2 cells were examined by western blotting. (E) Autophagosomes in LAD2 cells were observed by TEM. (F) An IF assay for LC3B expression in LAD2 cells. Magnification, ×400; scale bar = 10 μm. ELISA data represent changes in the concentrations of IL‐4 (G), IL‐5 (H), and IL‐13 (I) in culture supernatants. *p < .05; **p < .01; ***p < .001. ELISA, enzyme‐linked immunoassay; IL, interleukin; RORα, receptor related orphan receptor α; TEM, transmission electron microscope.

Additionally, we further explored the effects of RORα in AR HNEpC cells on the related functions of MCs. LAD2 cells were co‐cultured with HNEpC cells and treated as described above for 24 h. ELISA data showed that RORα overexpression in AR HNEpC cells could lead to remarkable decreases in β‐hexosaminidase, histamine, and tryptase concentrations, and RORα silencing in AR HNEpC cells could cause significant increases in the levels of those three compounds in the culture supernatants (Figure 7A–C). We also found that RORα overexpression in AR HNEpC cells reduced p62 expression, and increased LC3BII and Beclin 1 expression in LAD2 cells, while RORα silencing had the opposite effects on the expression of those three proteins (Figure 7D). Similarly, TEM results showed that RORα overexpression in AR HNEpC cells dramatically increased the numbers of autophagosomes, and RORα silencing in AR HNEpC cells notably decreased the numbers of autophagosomes in LAD2 cells (Figure 7E). IF data revealed that RORα overexpression in AR HNEpC cells upregulated LC3B expression, and RORα silencing in AR HNEpC cells markedly downregulated LC3B expression in LAD2 cells (Figure 7F). Additionally, ELISA data revealed that RORα overexpression in AR HNEpC cells markedly reduced IL‐4, IL‐5, and IL‐13 levels, and RORα silencing in AR HNEpC cells notably increased the levels of IL‐4, IL‐5, and IL‐13 in LAD2 cells (Figure 7G–I). When taken together, these data verified that RORα in AR HNEpC cells could prevent MC degranulation and inflammation, and accelerate autophagy in LAD2 cells.

### 3.8 RORα in AR HNEpC cells regulated MC degranulation, autophagy, and NLRP3‐induced inflammations via autophagy in LAD2 cells

**Figure 8:**
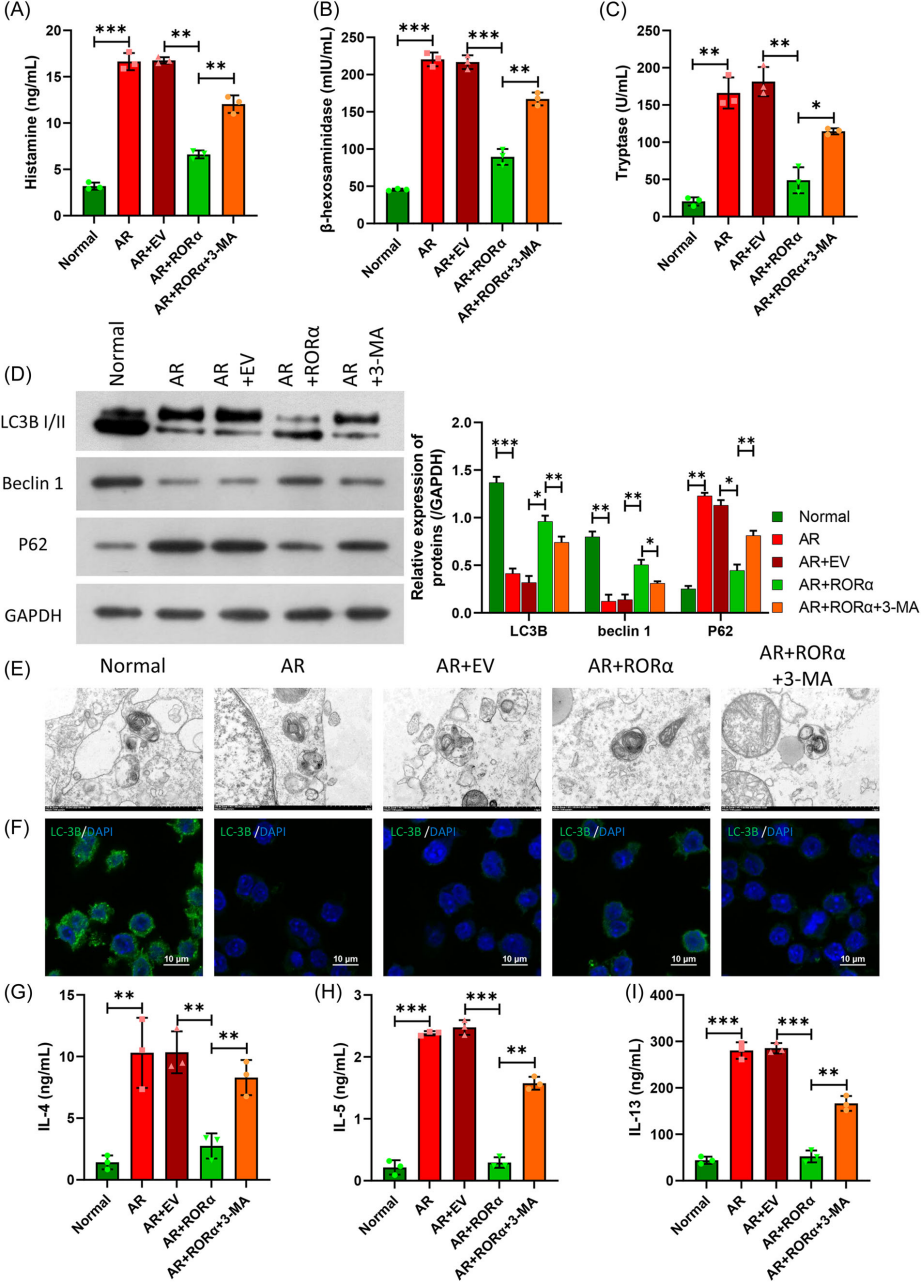
RORα in AR HNEpC cells regulated mast cell degranulation, autophagy, and NLRP3 inflammation via autophagy in LAD2 cells. AR HNEpC cells were treated with the RORα overexpression plasmid or/and 3‐MA, and then co‐cultured with LAD2 cells for 24 h. ELISA kits were used to assess changes in the levels of β‐hexosaminidase (A), histamine (B) and tryptase (C) in cell supernatants. (D) Western blotting was performed to detect changes in the expression of autophagy‐related proteins. (E) Changes in autophagosomes were detected by TEM. (F) Changes in LC3B expression were detected by IF assays. Magnification, ×400; scale bar = 10 μm. (G–I) The levels of inflammatory factors (IL‐4, IL‐5, and IL‐13) were evaluated by ELISA. *p < .05; **p < .01; ***p < .001. ELISA, enzyme‐linked immunoassay; IL, interleukin; RORα, receptor related orphan receptor α; TEM, transmission electron microscope.

We next conducted a rescue experiment to further verify whether autophagy is involved in the relevant functions of LAD2 cells mediated by RORα in AR HNEpC cells. As shown in Figure 8, the decreases in β‐hexosaminidase, histamine, and tryptase levels caused by RORα overexpression in AR HNEpC cells could be partially reversed by treatment with an autophagy inhibitor (3‐MA) (Figure 8A–C). Western blot results showed that the upregulation of LC3BII and Beclin 1, and downregulation of p62 mediated by RORα overexpression in AR HNEpC cells could be significantly attenuated by 3‐MA (Figure 8D). TEM results revealed that 3‐MA could significantly reduce autophagosome formation in LAD2 cells, while RORα overexpression increased autophagosome formation in AR HNEpC cells (Figure 8E). Meanwhile, IF results showed that 3‐MA could upregulate LC3B expression in LAD2 cells, which was significantly downregulated by RORα overexpression in AR HNEpC cells (Figure 8F). In addition, ELISA results indicated that 3‐MA could attenuate the reductions in IL‐4, IL‐5, and IL‐13 levels in LAD2 cells caused by RORα overexpression in AR HNEpC cells (Figure 8G–I). These results indicated that autophagy was crucial for MC degranulation, autophagy, and NLRP3‐induced inflammation in LAD2 cells that were inhibited by RORα in AR HNEpC cells.

### 3.9 Overexpression of RORα prevented MC degranulation, attenuated NLRP3‐induced inflammation, and induced autophagy in the AR model mice

**Figure 9:**
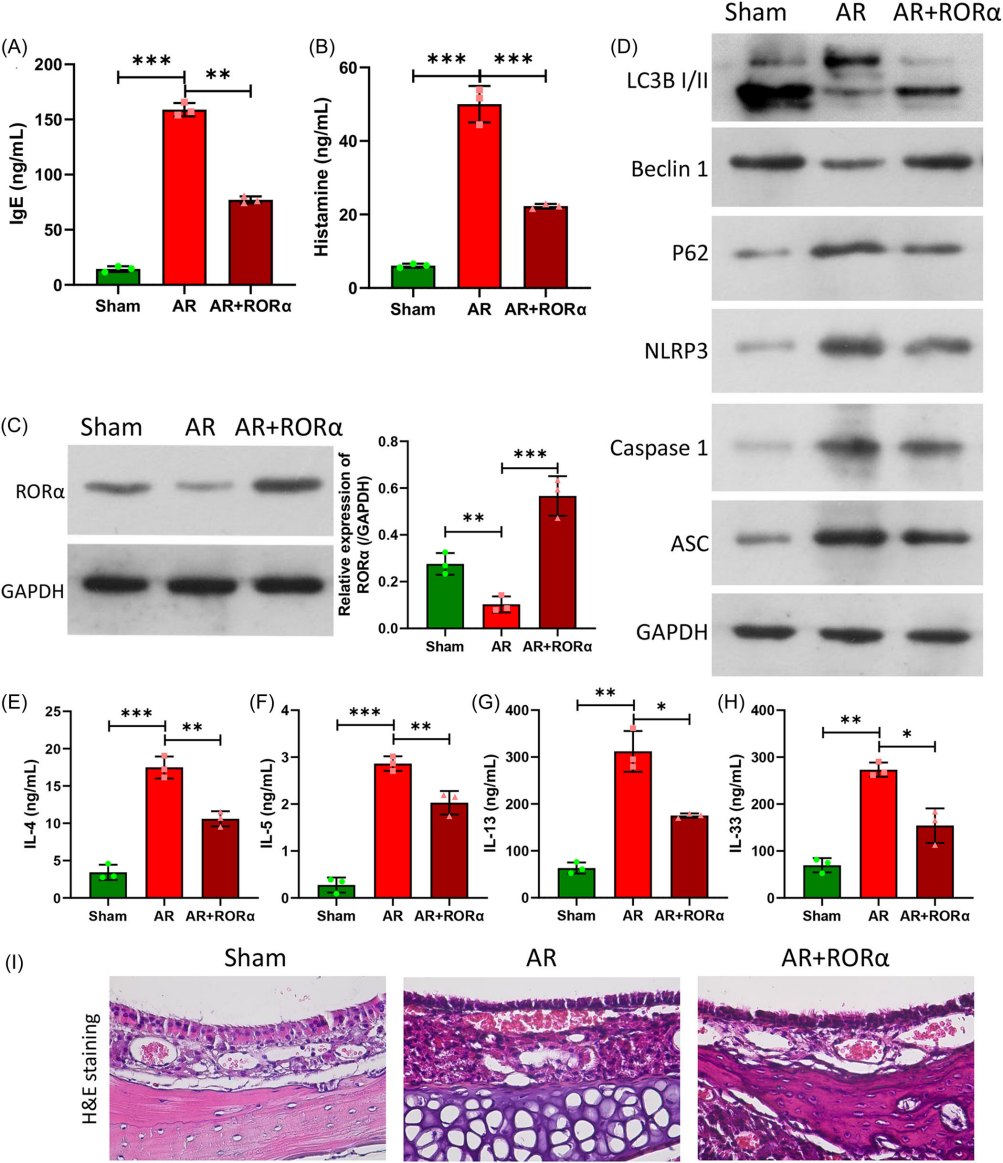
RORα attenuated NLRP3‐induced inflammation, and induced autophagy in the AR model mice. An AR mouse model was established, and the mice were injected with RORα expression plasmids. Changes in IgE (A) and histamine (B) levels were detected by ELISA. (C) Western blot results showed the changes in RORα expression. (D) The levels of autophagy‐related proteins (LC3BII, Beclin1, P62) and NLRP3 inflammation‐related proteins (NLRP3, Caspase 1, ASC) were determined by western blotting. The concentrations of IL‐4 (E), IL‐5 (F), IL‐13 (G), and IL‐33 (H) in mouse serum were determined by ELISA. (I) H&E staining showed changes in the pathological structure of nasal mucosal tissues. *p < .05; **p < .01; ***p < .001. AR, allergic rhinitis; ELISA, enzyme‐linked immunoassay; H&E, hematoxylin‐eosin; IgE, immunoglobulin E; IL, interleukin; RORα, receptor related orphan receptor α.

Based on the results of in vitro experiments, we further verified the role and mechanism of RORα in vivo in mice. Our experiments revealed that relative to the sham group, the levels of IgE and histamine were markedly increased in the AR model group, and RORα Adenovirus delivery by nebulization drug delivery could significantly decrease the levels of IgE and histamine in the AR model mice (Figure 9A,B). Meanwhile, we also verified that RORα Adenovirus could markedly upregulate RORα expression in the AR model mice (Figure 9C). Furthermore, our data showed that when compared to the sham group, LC3B and Beclin 1 were notably downregulated, and p62, NLRP3, Caspase 1, and ASC were significantly upregulated in the AR model mice, and those changes in protein levels could also be dramatically reversed by RORα Adenovirus (Figure 9D). Moreover, we verified that the increases in IL‐4, IL‐5, IL‐13, and IL‐33 levels in the AR model mice could be significantly attenuated by RORα Adenovirus (Figure 9E–H). H&E staining showed that the overall structure of the mouse nasal mucosa in the shame group was normal, with a uniform distribution of epithelial cells, and the numbers of infiltrated inflammatory cells in the submucosal tissue spaces were low. However, in the AR model group, epithelial defects in the nasal mucosa of mice were detected and were accompanied by large numbers of infiltrated inflammatory cells and decreased numbers of glands (Figure 9I). In the RORα Adenovirus group, the pathological changes in the nasal mucosa of AR tissue were significantly reduced (Figure 9D). These findings revealed the effects of RORα on autophagy, NLRP3‐induced inflammation, and allergic reactions in AR in vivo.

## 4 DISCUSSION

AR is a frequently diagnosed allergic disease in otolaryngology, and its prevalence among children ranges from 10% to 40%.^2^ At present, the pathogenesis of AR is not completely clear. Medications can relieve approximately 80% of AR symptoms in patients.^24^ However, AR is prone to multiple relapses, which creates certain obstacles to its clinical treatment. Therefore, it is extremely crucial to investigate the specific markers of AR and search for reliable and effective therapeutic targets. Derp1, as a critical allergen, is also often used to construct cellular models of AR. Based on previous studies,^25^ we induced HNEpC cells with Derp1 to construct AR cell models. Moreover, our results confirmed that Derp1 could induce inflammasome formation and the migration of HNEpC cells. In addition, an AR mouse model was established by treatment with OVA,^26^, ^27^ and all the treated mice exhibited obvious nose scratching, sneezing, and runny nose. The total score for the treated mice was >5, indicating that the model had been successfully established.

The histological features of AR include an enhanced Th2 inflammatory response, eosinophil infiltration, allergen‐specific IgE production, and basement membrane thickening.^5^ Activation of Th2 cells and the further release of cytokines induce the aggregation of various immune cells, leading to AR development.^7^ Recent study proved that Th17 cells could induce Th2 cell‐mediated eosinophilic airway inflammation in a mouse model of asthma.^28^ Moreover, MCs can migrate to sites of inflammation and initiate or induce inflammatory responses by secreting multiple proinflammatory mediators.^29^ An allergic reaction is a type I tachyphylactic hypersensitivity reaction mediated by IgE.^30^ In MCs, which are the preferred target cells of an allergic reaction, IgE can bind to its high affinity receptor FcsRI, and thereby put the organism in a sensitized state.^29^ MC degranulation is a defense response of the body and the basis for pathological reactions such as type I hypersensitivity and inflammation.^31^ In an allergic reaction, MCs release inflammatory mediators such as histamine, leukotrienes, bradykinin, and eosinophil chemokines, which can lead to increased vascular permeability, inflammatory cell infiltration, and allergic immunopathological responses such as congestion of the nasal mucosa and contraction of respiratory smooth muscle.^32^, ^33^ Therefore, blocking the migration of MCs is also a strategy for controlling inflammatory diseases. For example, Guo et al. has uncovered that estrogen, through the estrogen receptor, induces the expression of NLRP3, enabling the activation of NLRP3 inflammasome and the production of IL‐1β in MCs, which propels endometriosis development and fibrogenesis.^34^ In our study, we proved that the NLRP3 inflammasome was activated, and cell migration was markedly enhanced in the AR model cells, indicating that NLRP3 inflammasomes and cell migration are relevant to the AR process.

The immune inflammatory response plays a key role in AR progression.^35^ The NLRP3 inflammasome is an intracellular complex composed of NLRP3, ASC, and Caspase‐1,^36^ and NLRP3 plays a crucial role in promoting the inflammatory response.^36^ It has been suggested that NLRP3 inflammasome activation is involved in AR pathogenesis and associated with the degree of the inflammatory response.^37^ IL‐33, as a product of the NLRP3 inflammasome,^38^ has been reported to mediate immune responses via the specific receptor, ST2.^39^ Additionally, IL‐33 can accelerate the accumulation of various inflammatory cells, such as eosinophils, basophils, and MCs in the nasal mucosa, as well as the release of cytokines, such as IL‐4, IL‐5, and IL‐13, which further induce the development of AR.^40^, ^41^ Therefore, NLRP3 inflammasome activation enhanced IL‐33 might associated with AR progression. The upregulation of IL‐33 caused by the activation of the NLRP3 inflammasome may be a key factor triggering the onset and progression of AR.

RORα has been reported to play a key role in the differentiation of Th17 cells. RORα, as a transcription factor, is also essential for the development of ILC2 cells,^20^ which plays a proinflammatory role in the pathogenesis of AR,^42^ and another recent study revealed that the RORα‐related pathway is associated with the type II innate lymphocyte response in AR patients.^43^ RORα can intervene in the occurrence of inflammatory responses through complex pathways. Gao et al.^44^ has found that RORα, through the SIRT1 molecule, can inhibit the nuclear factor kappa B (NF‐κB) pathway, thus playing an anti‐inflammatory role in inflammatory bowel disease. Therefore, we hypothesized that RORα might exert an inhibitory effect on the NLRP3 inflammasome by suppressing the NF‐κB pathway. In our study, we found that overexpression of RORα could prevent NLRP3 inflammasome activation in AR HNEpC cells and the migration of those cells.

Autophagy is a special mode of programmed cell death in eukaryotes, and plays a crucial role in biological processes.^45^, ^46^ LC3 is one of the key proteins in the autophagy process. During autophagy, LC3I binds to phosphatidylethanolamine to form LC3II, and an intact autophagosome. The amount of LC3II in the body is closely related to autophagy activity and can be used as a marker molecule to measure the degree of autophagy.^47^ Autophagy is closely associated with AR, which may be related to its involvement in MC degranulation. On the one hand, autophagy promotes allergic reactions by facilitating MC degranulation and inflammatory responses. For example, Li et al.^48^ showed that inhibition of MC degranulation significantly reduced its degranulation. On the other hand, it has also been found that further enhancement of apoptosis by promoting autophagy may still inhibit MC action.^49^ Additionally, a complex interplay exists between IL‐33 and autophagy. For instance, in models of colitis, IL‐33 has been shown to promote autophagy occurrence in macrophages.^50^ Besides, in models of traumatic brain injury, IL‐33 has been demonstrated to inhibit the occurrence of autophagy and modulate brain injury.^51^ In our study, In this study, we found that NLRP3 inflammasome‐mediated IL‐33 may serve as a link between nasal epithelial cells and MCs.

## 5 CONCLUSIONS

**Figure 10:**
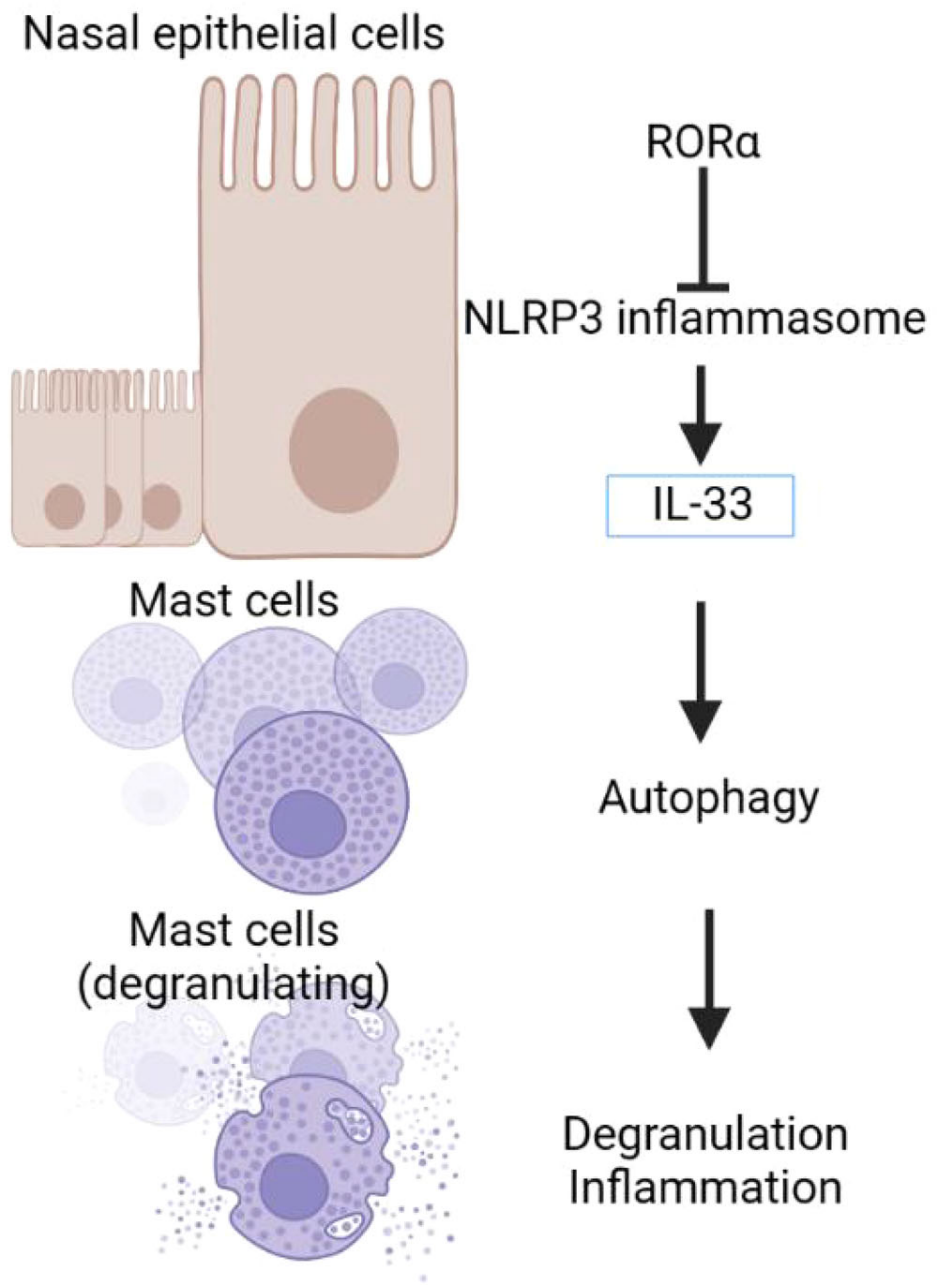
Graphic summary of this study.

In summary, our data showed that RORα overexpression could inhibit IL‐33 expression, NLRP3 inflammasome activity, and cell migration in AR model cells, and also attenuate MC degranulation and inflammation via autophagy (Figure 10). These findings suggest RORα as a target for AR therapy.

## AUTHOR CONTRIBUTIONS

**Wangbo Yu**: Methodology; Resources; Software; Writing—original draft. **Jingwei Du**: Conceptualization; Data curation; Methodology; Resources; Validation; Visualization; Writing—original draft. **Lijuan Peng**: Conceptualization; Data curation; Formal analysis; Investigation; Methodology; Resources.

## Data Availability

Data will be provided based on reasonable request.
